# A novel *PKHD1* splicing variant identified in a fetus with autosomal recessive polycystic kidney disease

**DOI:** 10.3389/fgene.2023.1207772

**Published:** 2023-06-29

**Authors:** Mingzhu Miao, Liqun Feng, Jue Wang, Cheng Xu, Xiaotian Su, Guoying Zhang, Shoulian Lu

**Affiliations:** ^1^Department of Obstetrics and Gynecology, The First Affiliated Hospital of Nanjing Medical University, Nanjing, China; ^2^Department of Pathology, The First Affiliated Hospital of Nanjing Medical University, Nanjing, China; ^3^ Department of Bioinformatics, Berry Genomics Co., Ltd., Beijing, China

**Keywords:** autosomal recessive polycystic kidney disease, PKHD1, whole-exome sequencing, intronic variant, minigene, aberrant splicing

## Abstract

**Objective:** Variants of the polycystic kidney and hepatic disease 1 (*PKHD1*) gene are associated with autosomal recessive polycystic kidney disease (ARPKD). This study aimed to identify the genetic causes in a Chinese pedigree with ARPKD and design a minigene construct of the *PKHD1* gene to investigate the impact of its variants on splicing.

**Methods:** Umbilical cord samples from the proband and peripheral blood samples from his parents were collected, and genomic DNA was extracted for whole-exome sequencing (WES). Bioinformatic analysis was used to identify potential genetic causes, and Sanger sequencing confirmed the existence of variants within the pedigree. A minigene assay was performed to validate the effects of an intronic variant on mRNA splicing.

**Results:** Two variants, c.9455del (p.N3152Tfs*10) and c.2408-13C>G, were identified in the *PKHD1* gene (NM_138694.4) by WES; the latter has not been previously reported. In silico analysis predicted that this intronic variant is potentially pathogenic. Bioinformatic splice prediction tools revealed that the variant is likely to strongly impact splice site function. An *in vitro* minigene assay revealed that c.2408-13C>G can cause aberrant splicing, resulting in the retention of 12 bp of intron 23.

**Conclusion:** A novel pathogenic variant of *PKHD1*, c.2408-13C>G, was found in a fetus with ARPKD, which enriches the variant spectrum of the *PKHD1* gene and provides a basis for genetic counseling and the diagnosis of ARPKD. Minigenes are optimal to determine whether intron variants can cause aberrant splicing.

## 1 Introduction

Autosomal recessive polycystic kidney disease (ARPKD) (OMIM #263200), also known as infantile polycystic kidney disease, is a rare and serious fibrocystic disease of the liver and kidneys that usually occurs in fetuses or neonates (Guay-Woodford et al., 1995). The incidence of ARPKD is estimated to be approximately 1 per 10,000–40,000 live births, with an average incidence of approximately 1 in 20,000 (Bergmann, 2017), which is the same in males and females (Bergmann et al., 2004b). The clinical phenotype of ARPKD varies greatly (Bergmann et al., 2018). Prenatal presentations are mainly characterized by massively enlarged, echogenic kidneys, oligohydramnios and the “Potter” sequence with pulmonary hypoplasia, Potter facies, and spinal and limb abnormalities caused by oligohydramnios (Guay-Woodford and Desmond, 2003), which lead to respiratory insufficiency and perinatal death in approximately 30% of the affected newborns (Bergmann et al., 2005b). Most surviving patients progress to end-stage renal disease (ESRD) at various ages. Patients also develop liver diseases, including dilated biliary ducts, congenital hepatic fibrosis, and portal hypertension (Caroli disease) (Gunay-Aygun et al., 2006; Hartung and Guay-Woodford, 2014). The prognosis for these patients is poor.

Variants in the polycystic kidney and hepatic disease 1 (*PKHD1*) gene have been identified as the main genetic basis of ARPKD (Onuchic et al., 2002; Ward et al., 2002). The *PKHD1* gene is located on human chromosome 6p12.3-p12.2, and contains 86 coding exons (Onuchic et al., 2002; Ward et al., 2002; Bergmann et al., 2004a; Gross, 2016), with the largest reading frame encompassing 67 exons (NM_138694.3); however, multiple alternatively spliced transcripts have been described (Bergmann et al., 2004a). *PKHD1* is expressed at moderate levels in the adult kidney, pancreas, and fetal kidney, with lower expressions in the liver. There is almost no expression in the peripheral blood. Fibrocystin is a large protein with receptor-like properties (Onuchic et al., 2002; Ward et al., 2002). It is a receptor protein that acts on the collecting duct and promotes biliary differentiation. Along with polycystin-1 and polycystin-2, fibrocystin has been shown to interact at the molecular level, in addition to direct interactions with protein products. To date, more than 500 *PKHD1* variants have been reported; their variant types are diverse, including missense, nonsense, splicing, insertions, and deletions (Sweeney and Avner, 1993-2019; Adeva et al., 2006).

In this study, we used whole-exome sequencing (WES) to evaluate the genetic causes of suspected ARPKD in a Chinese fetus. We identified two variants in the *PKHD1* gene in the fetus, one of which was a novel variant located in an intron. To determine whether this site was pathogenic to the affected fetus, we performed a *PKHD1* variant pathogenicity study. A minigene splicing assay (Bonnet et al., 2008; Thery et al., 2011) was conducted to identify the novel splice-altering variant of *PKHD1*. The contribution of this variant to the etiology of ARPKD was evaluated.

## 2 Materials and methods

### 2.1 Clinical data

**FIGURE 1:**
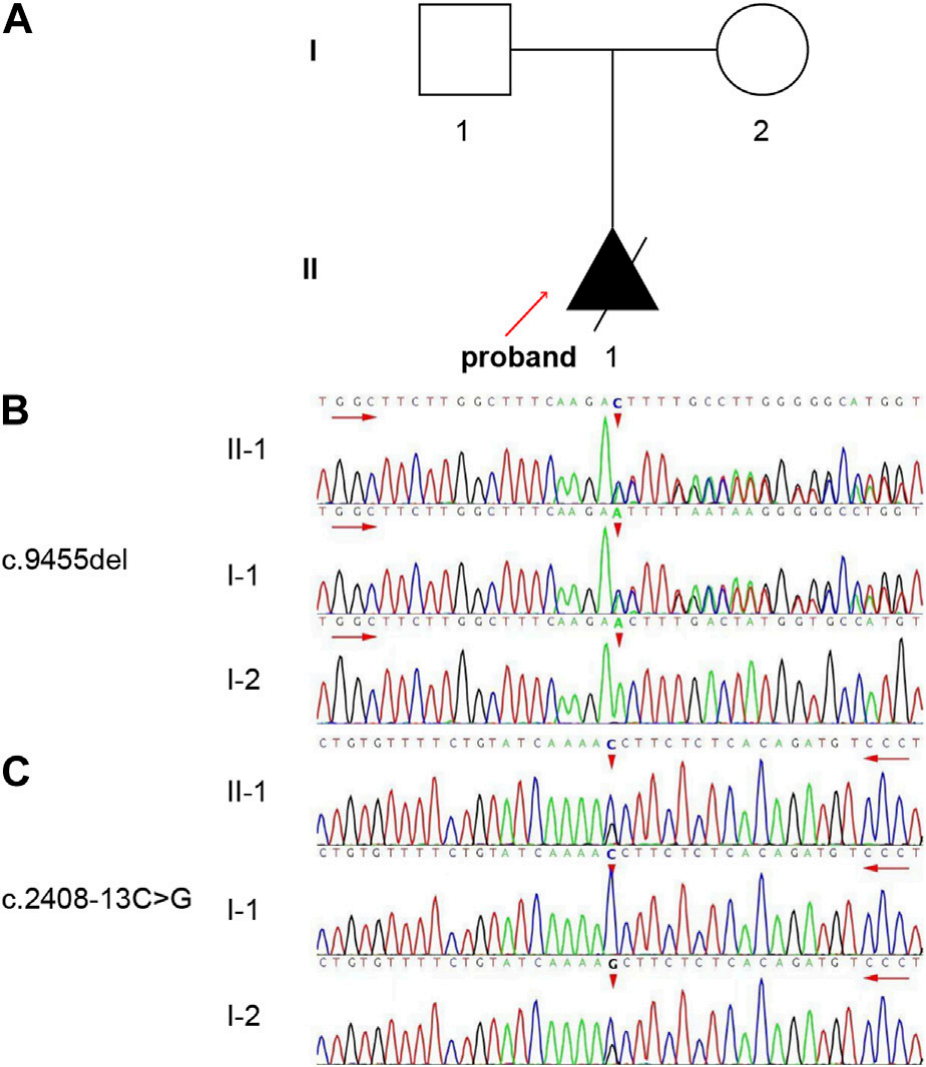
The pedigree and genotype of the family. **(A)** One family member in this pedigree was diagnosed with ARPKD. The solid triangle with an arrow indicates the proband (II-1). **(B, C)** Validation of the variants by Sanger sequencing. The short red arrows indicate the variant sites [**(B)** c.9455del, **(C)** c.2408-13C>G]. The long red arrows indicate the sequencing direction.

A Chinese family member was recruited from The First Affiliated Hospital of Nanjing Medical University, Nanjing, China. The family pedigree is shown in Figure 1A. The proband (II -1) was a fetus suspected of having ARPKD based on the prenatal ultrasound. The father (I-1) and mother (I-2) of the proband requested pregnancy termination at 30 weeks and 2 days of gestation. The proband (II -1) was subsequently aborted because of the poor prognosis. The proband was male, and his parents had normal kidneys and livers. Clinical data collection, imaging examinations, fetal autopsies, and genetic analyses were performed at our hospital. This study was approved by the Ethics Committee of First Affiliated Hospital of Nanjing Medical University (approval number: 2022-QT-09). Written informed consent was obtained from the parents of the patient.

### 2.2 Whole-exome sequencing (WES) and bioinformatic analysis

Umbilical cord samples from the proband and peripheral blood samples (2-3 mL, EDTA anticoagulant) from his parents were collected. Total genomic DNA was extracted from all the participants using a QIAamp DNA extraction kit (Qiagen GmbH, Hilden, Germany) according to the manufacturer’s protocol and sent to Berry Genomics (Beijing, China) for WES. The Novaseq6000 platform (Illumina, San Diego, CA, United States), with 150 bp paired-end sequencing, was used to sequence the genomic DNA of the family. More than 98% of the targeted regions were examined at depths greater than 20×.

Raw image files were processed using CASAVA v1.82 (Berry Genomics) for base calling and raw data generation. The sequencing reads were aligned to the human reference genome (hg38/GRCh38) using the Burrows-Wheeler Aligner tool (Abuín et al., 2015). PCR duplicates were removed using Picard v1.57 (http://picard.sourceforge.net/). The Verita Trekker^®^ Variants Detection System (Berry Genomics) and third-party software GATK (https://software.broadinstitute.org/gatk/) were employed for variant calling. Variant annotation and interpretation were conducted using ANNOVAR (Wang et al., 2010) and the Enliven Variant Annotation Interpretation System authorized by Berry Genomics. Once a variant was considered as the etiology of a recessive disorder, manual inspection for coverage and additional variants of the entire coding domain was performed using Integrated Genomics Viewer (Robinson et al., 2011; Thorvaldsdóttir et al., 2013).

### 2.3 Variant validation analysis and co-segregation analysis

The variants in the *PKHD1* gene (NM_138694.4) identified using WES were confirmed by Sanger sequencing and co-segregation analysis. Sanger sequencing was performed on an ABI 3730XL DNA Sequencer (Applied Biosystems, Thermo Fisher Scientific, Waltham, MA, United States). The sequences were aligned to the reference sequence using the Mutation Surveyor software (Dong and Yu, 2011; Minton et al., 2011). Segregation analysis was performed on the family members in this study and the literature (Cao et al., 2019).

### 2.4 In silico analysis

The potential effect on splicing was predicted with three splice-site prediction programs: Human Splicing Finder (HSF) version 3.1 (http://www.umd.be/HSF3/HSF.shtml), Varseak (https://varseak.bio/index.php), and SpliceAI (https://spliceailookup.broadinstitute.org/).

### 2.5 Minigene splicing assay

#### 2.5.1 Construction of recombinant vectors

Two groups of recombinant vectors were constructed: pcMINI-C-*PKHD1*-wild type (wt)/mutant type (mut) and pcDNA3.1-*PKHD1*-wt/mut. Two rounds of PCR were performed using nested primers. The first PCR was performed using genomic DNA as a template, with *PKHD1*-35726-F and *PKHD1*-40380-R as the primers, for 30 cycles, and the second PCR was performed using the products from the first round of PCR as a template, with *PKHD1*-36009-F and *PKHD1*-40093-R as the primers, for 30 cycles. For pcMINI-C-*PKHD1*-wt and pcDNA3.1-*PKHD1*-wt, PCR was performed using the PCR products from the second round of nested PCR as a template, with pcMINI-C-*PKHD1*-BamHI-F/pcMINI-C-*PKHD1*-XhoI-R and pcDNA3.1-*PKHD1*-BamHI-F/pcDNA3.1-*PKHD1*-XhoI-R as the primers, for 30 cycles. For pcMINI-C-*PKHD1*-mut, PCR was performed using PCR products from the second round nested PCR as a template, with pcMINI-C-*PKHD1*-BamHI-F and *PKHD1*-mut-R as the primers to amplify the left half of this recombinant vector, for 30 cycles, and with *PKHD1*-mut-F and pcMINI-C-*PKHD1*-XhoI-R as the primers to amplify the right half, for 30 cycles; the last round of PCR was performed using a 1:1 mixture of the left and right half as a template, with pcMINI-C-*PKHD1*-BamHI-F and pcMINI-C-*PKHD1*-XhoI-R as the primers, for 30 cycles. For pcDNA3.1-*PKHD1*-mut, the template and amplification procedures were the same as those for pcMINI-C-*PKHD1*-mut, except that the primers used were pcDNA3.1-*PKHD1*-BamHI-F, *PKHD1*-mut-R, *PKHD1*-mut-F, and pcDNA3.1-*PKHD1*-XhoI-R (the genomic DNA and gene names are shown in Table 1, and the primers for constructing the minigene vectors are listed in Table 2). Electrophoresis and gel recovery of the final round of the PCR products were performed. The amplified lengths of pcMINI-C-*PKHD1*-wt and pcMINI-C-*PKHD1*-mut were 1,858 bp. The amplified lengths of pcDNA3.1-*PKHD1*-wt and pcDNA3.1-*PKHD1*-mut were 2,641 bp.

**TABLE 1:** Genomic DNA and gene names corresponding to each group.

Genomic DNA	Name of gene
Control group-1	pcMINI-C-*PKHD1*-wt (wild-type)
Experimental group-1	pcMINI-C-*PKHD1*-mut (c.2408-13C>G)
Control group-2	pcDNA3.1-*PKHD1*-wt (wild-type)
Experimental group-2	pcDNA3.1-*PKHD1*-mut (c.2408-13C>G)

**TABLE 2:** The primers for constructing the minigene vectors.

Primers	Sequences (5′ to 3′)
*PKHD1*-35726-F	Ggt​tgc​aca​atg​atg​aga​ct
*PKHD1*-36009-F	cag​aga​gga​agc​cca​cca​ta
*PKHD1*-40093-R	Ggg​aac​tgt​aca​tac​cct​gt
*PKHD1*-40380-R	Tca​gtt​tgg​gtc​ccc​agg​tg
pcMINI-C-*PKHD1*-BamHI-F	GCT​CGG​ATC​Cag​cta​atg​aaa​tgg​gaa​ctt
pcMINI-C-*PKHD1*-XhoI-R	TAG​ACT​CGA​GCT​GAG​TAT​GCT​GGT​TGG​CAG
pcDNA3.1-*PKHD1*-BamHI-F	GCT​CGG​ATC​CAT​Gcg​CTC​TGT​GCC​CAC​TGA​AGG​AA
pcDNA3.1-*PKHD1*-XhoI-R	TAG​ACT​CGA​GCC​TGA​TAA​AAT​TGG​GCA​AAT
*PKHD1*-mut-F	ttt​tct​gta​tca​aaa​Gct​tct​ctc​aca​gAT​G
*PKHD1*-mut-R	CAT​ctg​tga​gag​aag​Ctt​ttg​ata​cag​aaa​a
pcDNA3.1-F	CTA​GAG​AAC​CCA​CTG​CTT​AC

The PCR products, pcMINI-C-*PKHD1*-wt and pcMINI-C-*PKHD1*-mut, were purified and inserted into the eukaryotic expression vector pcMINI-C using BamHI/XhoI to construct two sets of plasmids: pcMINI-C-*PKHD1*-wt and pcMINI-C-*PKHD1*-mut. The PCR products pcDNA3.1-*PKHD1*-wt and pcDNA3.1-*PKHD1*-mut were purified and inserted into the eukaryotic expression vector pcDNA3.1 using BamHI/XhoI to construct two sets of plasmids: pcDNA3.1-*PKHD1*-wt and pcDNA3.1-*PKHD1*-mut. The recombinant plasmids were digested with BamHI and XhoI and verified by gene sequencing.

The pcDNA3.1 vector was constructed using exon 23 (128 bp)-intron 23 (2,303 bp)-and exon 24 (185 bp). The sequence was constructed using two complete exons of *PKHD1* and an intron between them. The pcMINI-C vector contained the general exon A and intron A. During construction, intron 23 (615 bp)-exon 24 (185 bp)-intron 24 (915 bp)-and exon 25 (123 bp) were inserted into the pcMINI-C vector to form exon A-intron A-intron 23 (615 bp)-exon 24 (185 bp)-intron 24 (915 bp)-exon 25 (123 bp). Intron 23 intercepted the part of the sequence that overlapped with intron A to simulate the complete intron sequence. The pcMINI-C vector contained three exons: exon A, exon 24, and exon 25. The pcMINI-C vector inserted intron 24 and exon 25 into the vector to determine whether the variant led to the overall skipping of exon 24.

#### 2.5.2 Transfection of eukaryotic cells

This experiment was designed to use two kinds of cells for transfection [human embryonic kidney cells (HEK293T; ATCC) and cervical cancer cells (HeLa; ATCC)]. HEK293T and HeLa cells were cultured in Dulbecco’s Modified Eagle’s Medium (DMEM; Gibco, #11965092) supplemented with 10% fetal bovine serum and 1% penicillin/streptomycin (PenStrep) at 37°C in a 5% CO_2_ humidified atmosphere. The cell pellet was resuspended in complete DMEM without PenStrep and thereafter the cells were plated in 6-well plates (1×10^6^ cells/well) and incubated until approximately 60% confluence before transfection. For each well, 3 µg recombinant plasmid DNA of either wt or mut was diluted with 250 µL OPTI-MEM (Gibco, #31985-070) and mixed well. Similarly, 8 µL of Liposomal Transfection Reagent was mixed with 250 µL OPTI-MEM and incubated at room temperature (20°C-25°C) for 5 min. The diluted plasmid solutions and Liposomal Transfection Reagent were mixed and incubated at room temperature for 20 min to form a DNA-Liposome complex. Thereafter, 500 µL/well of the mixture was added to 6-well plates of HEK293T and HeLa cells. The transfected cells were cultured at 37 °C in a 5% CO_2_ humidified atmosphere, and harvested at 48 h post-transfection for analysis.

#### 2.5.3 Reverse transcription-polymerase chain reaction (RT-PCR)

Total RNA was extracted from the HEK293T and HeLa cells using the TRIzol reagent (RNAiso PLUS, TaKaRa Bio Inc., Shiga, Japan). RNA concentration and purity were assessed using UV spectrophotometry, and the cDNA was synthesized using HifairTM 1st Strand cDNA Synthesis SuperMix for qPCR (gDNA digester plus, YEASEN Biotechnology, Shanghai, China). The PCR products were identified using 2% agarose gel electrophoresis and verified by sequencing.

## 3 Results

### 3.1 Clinical findings

**FIGURE 2:**
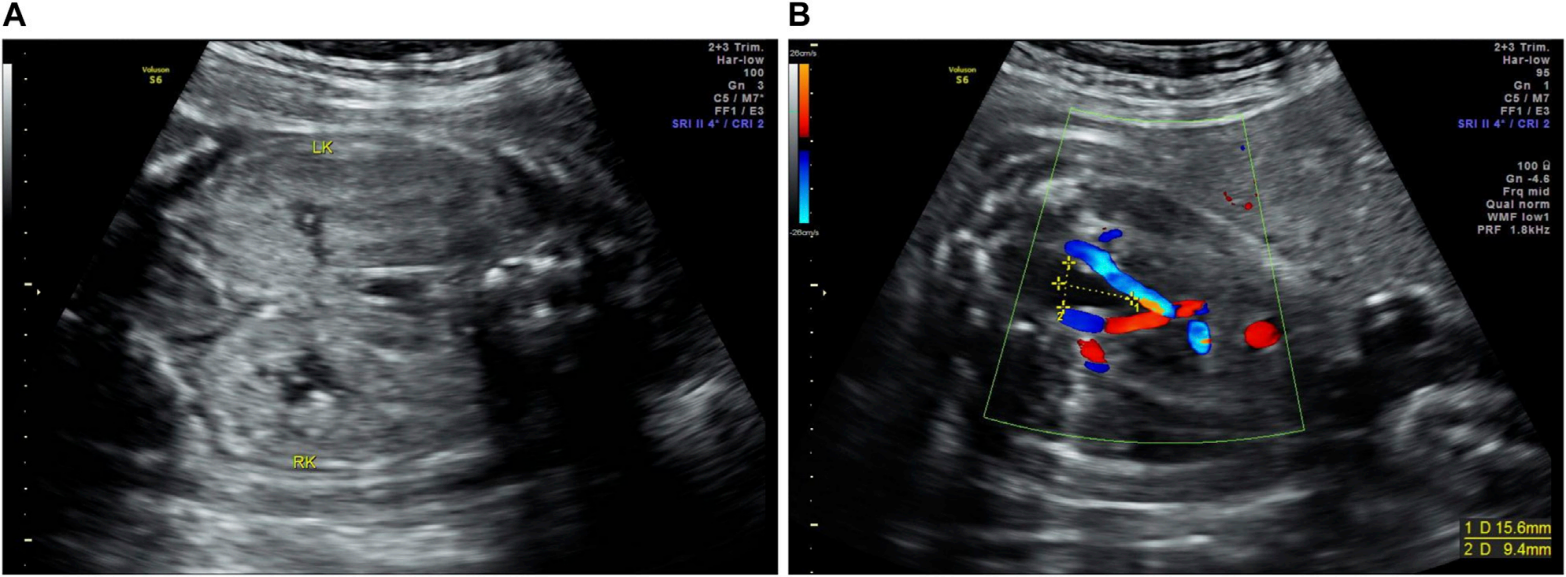
Prenatal ultrasound images of the proband (II-1). **(A)** The massively enlarged, echogenic kidneys of the proband (LK, left kidney; RK, right kidney). **(B)** The size of the bladder of the proband was 15.6 mm × 9.4 mm.

The proband (II-1) was diagnosed with suspected ARPKD at a gestational age of 30 weeks in our hospital via a prenatal ultrasound, which suggested massively enlarged, echogenic kidneys and oligohydramnios (Figure 2). The parents had normal kidneys and liver according to the abdominal ultrasound. When asked about their medical history at their local hospital, oligohydramnios [amniotic fluid index (AFI), 48 mm)] was detected via ultrasound at 16 weeks and 5 days of pregnancy. Oligohydramnios (AFI, 44 mm) was detected by ultrasonography at 25 weeks and 2 days of pregnancy, and both kidneys were slightly enlarged and echogenic. This was the couple’s first pregnancy; the couple was not consanguineous and denied a family history of polycystic kidney disease (PKD). After being fully informed of the prognosis of the fetus and genetic counseling, the couple requested pregnancy termination at 30 weeks and 2 days of gestation. An autopsy and genetic testing were performed to explore the etiology of fetal PKD. The autopsy of the aborted fetus showed that the bilateral kidneys were enlarged; the size of the left kidney was 5.8 cm × 4.1 cm × 2.0 cm, the size of the right kidney was 6.0 cm × 4.0 cm × 2.0 cm, and there were small cystic cavities in both kidneys. Microscopically, dilated tubular structures could be seen between the renal parenchyma, lined with cuboidal epithelium, and part of the epithelium was exfoliated and showed polycystic changes; uninvolved nephrons, cortical medulla structures, glomeruli, and renal tubules could be seen between the cysts. The size of the liver was 10.0 cm × 9.0 cm × 3.0 cm. Microscopically, the structure of the hepatic lobule did exist; the portal area could be observed, and no definite abnormalities were visible.

### 3.2 Genetic test results

Trio-WES was performed on the proband and his parents. Two variants were detected in the *PKHD1* and were confirmed by Sanger sequencing (Figures 1B, C). Compound heterozygous variants c.9455del: p.N3152Tfs*10 in exon 58 and c.2408-13C>G in intron 23 were identified. In addition, the father was a heterozygous carrier of the c.9455del variant (Figure 1B) and the mother was a heterozygous carrier of the c.2408-13C>G variant (Figure 1C).

According to the “Interpretation Criteria and Guidelines for Gene Sequence Variation” established in 2015 (Richards et al., 2015), the pathogenicity analysis of c.9455del was carried out as follows: (1) the variant caused changes in the open reading frame of the gene, resulting in changes in the protein function (very strong pathogenic evidence, PVS1); (2) the variant was not found in gnomAD, the 1000 Genomes Project, and the ExAC dataset (medium strong pathogenic evidence, PM2); (3) it was reported that the phenotype of PKD was co-segregated with the genotype in two affected family members (Cao et al., 2019) (supportive pathogenic evidence, PP1); and (4) the clinical manifestations of the proband were highly consistent with the phenotype of ARPKD induced by the *PKHD1* gene variant (supportive pathogenic evidence, PP4). Taken together, the evidence intensity of the c.9455del variant was “PVS1+PM2+PP1+PP4” and was judged to be a pathogenic variant (very strong pathogenic evidence).

As suggested by the American College of Medical Genetics and Genomics (ACMG) and the Association for Molecular Pathology (AMP) guidelines and the expert specifications of variant interpretation guidelines for PKD (Guay-Woodford et al., 2014; Richards et al., 2015; Horie et al., 2016; Writing Group For Practice Guidelines For Diagnosis And Treatment Of Genetic Diseases Medical Genetics Branch Of Chinese Medical Association et al., 2020); the pathogenicity analysis of c.2408-13C>G was performed as follows: (1) the variant was not found in gnomAD, the 1000 Genomes Project, and the ExAC dataset (moderate pathogenic evidence, PM2); (2) the affected fetus was found to carry the *PKHD1* c.2408-13C>G and c.9455del compound heterozygous variants (moderate pathogenic evidence, PM3); (3) the splicing prediction software spliceAI indicated that the variant was likely to affect the splicing (supporting pathogenic evidence, PP3); and (4) the fetal phenotype, massively enlarged, echogenic kidneys, oligohydramnios, and autopsy results were highly specific to the *PKHD1* gene (supporting pathogenic evidence, PP4). Taken together, the evidence for the c.2408-13C>G variant was “PM2+PM3+PP3+PP4” and was judged to be a likely pathogenic variant.

### 3.3 In silico splicing analysis

**FIGURE 3:**
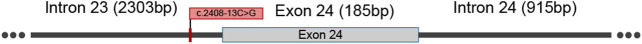
Schematic diagram of the location of the intronic variant (c.2408-13C>G) in the *PKHD1* gene. The variant is located at the 13th-to-last base of intron 23.

The c.2408-13C>G variant identified in *PKHD1* was located on the 13th-to-last base of intron 23 (Figure 3). According to the influence of mutation location on splicing from large to small, mutations are divided into four categories: class Ⅰ (classical region: exon ±1 or 2 bp), class Ⅱ (boundary region: exon 3 bp ∼ intron 6 bp and intron 12 bp ∼ exon 2 bp, not contain exon ±1 or 2 bp), class Ⅲ (exon interior region), and class Ⅳ (deep intron region) (Cartegni et al., 2002). This intronic variant was located in the deep intron and belonged to the “class IV mutation region” which affects splicing. Therefore, we used HSF, Varseak, and SpliceAI to predict changes in the splice sites caused by the variant. HSF predicted that the score of the wt acceptor site would decrease by 37.37% after the introduction of the variant. According to Varseak, the score of the wt acceptor site would decrease by 29.90% after the introduction of the variant. SpliceAI predicted that the wt acceptor site score would decrease by 0.67, and a new acceptor site could be produced with a score as high as 0.91. In summary, all the splicing prediction software programs indicated that the wt acceptor site would be broken or altered after the variant, suggesting that the variant (c.2408-13C>G) most probably affects splicing (Table 3).

**TABLE 3:** In silico splicing analysis of the *PKHD1* variant.

Gene	Variant	SpliceAI		Varseak		HSF	
		Type	Δ Score	Type	Score	Type	Score
*PKHD1*	c.2408-13C>G	wt Acceptor Site Loss	0.67	wt Acceptor Site	+26.83% to −3.08% (Δ Score: −29.90%)	wt Acceptor Site	7.76 to 4.86 (Δ Score: −37.37%)
		New Acceptor Site Gain	0.91	New Acceptor Site	no AG to +6.02%		

“Δ Score” indicates the delta score value before and after the variant. According to SpliceAI (https://spliceailookup.broadinstitute.org/), the wt acceptor site score loss was 0.67, and a new acceptor site could be produced with a score gain of 0.91. According to Varseak (https://varseak.bio/index.php), the score of the wt acceptor site would change from +26.83% to −3.08%, decreasing by 29.90%, and a new acceptor site could be produced with a score from no AG to +6.02%. According to HSF (http://www.umd.be/HSF3/HSF.shtml), the score of the wt acceptor site would change from 7.76 to 4.86, decreasing by 37.37%.

### 3.4 Results of the minigene assay

#### 3.4.1 Construction of recombinant vectors

**FIGURE 4:**
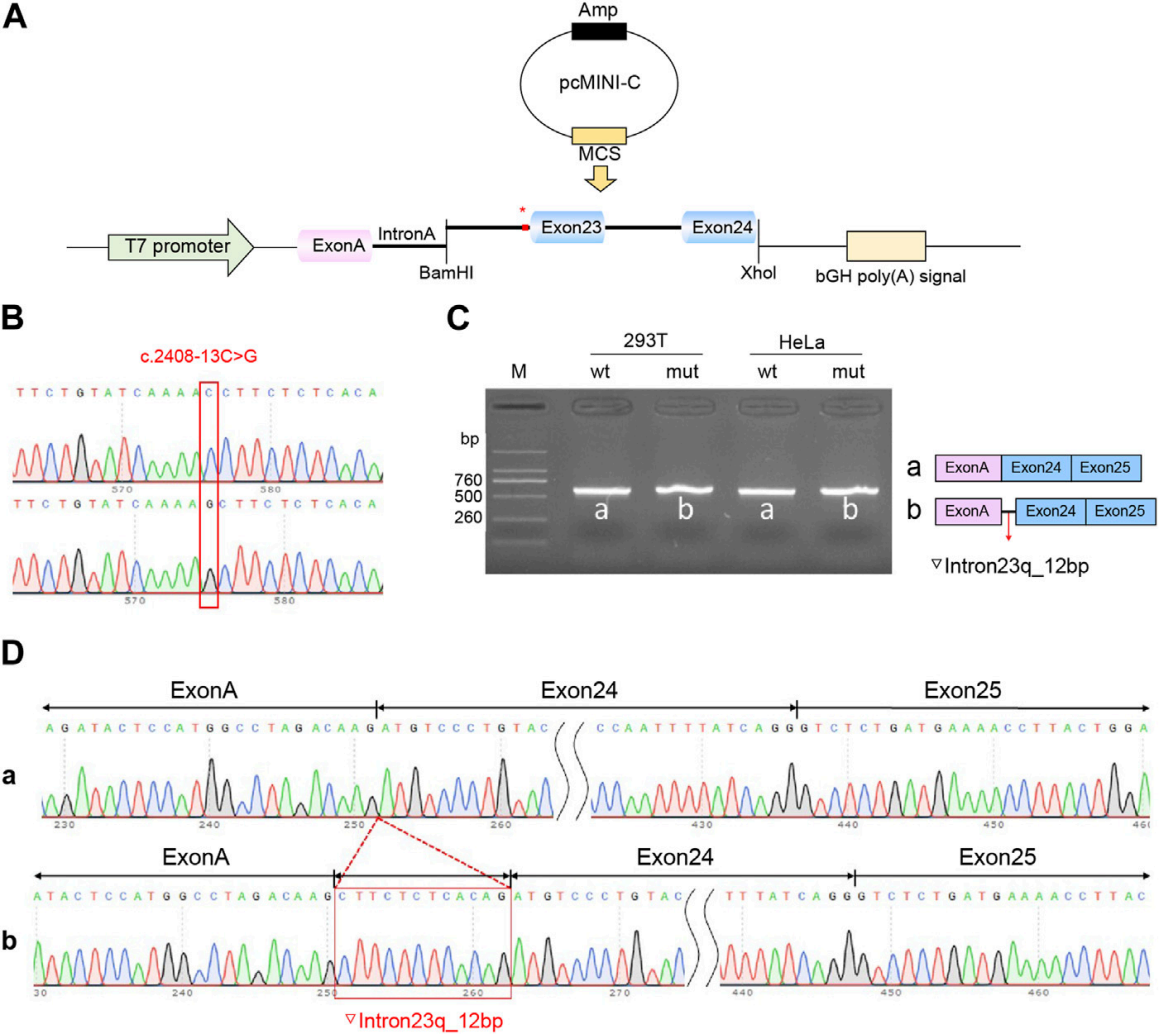
Splicing alteration was identified by a minigene assay using a pcMINI-C vector. **(A)** Schematic diagram of the minigene and vector construction. **(B)** Sequencing in the recombinant vector. The top of Figure 4B indicates the results of wt minigene sequencing, and the bottom shows the sequencing of the mut (c.2408-13C>G) minigene. Both are partial sequencing results. The red frame indicates the base changed by the variant. **(C)** Reverse-transcription polymerase chain reaction (RT-PCR) products were separated by electrophoresis of the pcMINI-*PKHD1*-wt/mut vector in 293T and HeLa cells. The different splicing products for wild-type (wt lane, 601 bp) and mutant-type (mut lane, 613 bp) are shown on 2% agarose gel electrophoresis and represented graphically. **(D)** The sequencing results for the bands.

**FIGURE 5:**
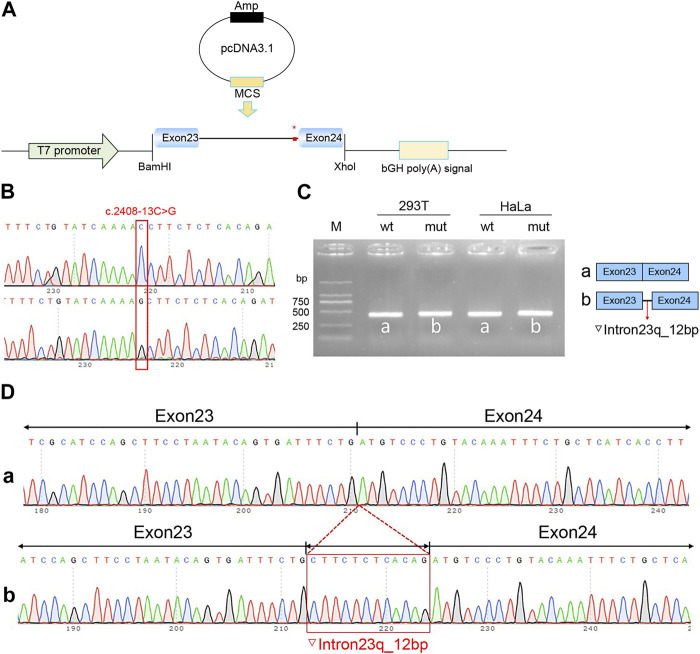
Splicing alteration was identified by a minigene assay using a pcDNA3.1 vector. **(A)** Schematic diagram of the minigene and vector construction. **(B)** Sequencing in the recombinant vector. The top of Figure 5B indicates the results of wt minigene sequencing, and the bottom shows the sequencing of the mut (c.2408-13C>G) minigene. Both are partial sequencing results. The red frame indicates the base changed by the variant. **(C)** Reverse-transcription polymerase chain reaction (RT-PCR) products were separated by electrophoresis of the pcDNA3.1-*PKHD1*-wt/mut vector in 293T and HeLa cells. The different splicing products for wild-type (wt lane, 434 bp) and mutant-type (mut lane, 446 bp) are shown on 2% agarose gel electrophoresis and represented graphically. **(D)** The sequencing results for the bands.

Recombinant plasmids pcMINI-C-*PKHD1*-wt, pcMINI-C-*PKHD1*-mut, pcDNA3.1-*PKHD1*-wt, and pcDNA3.1-*PKHD1*-mut were successfully constructed. The four recombinant plasmids were digested with restriction endonucleases XhoI and BamHI; the fragments were consistent with the expected fragments, confirming that the target fragment, the *PKHD1*-wt/mut minigene, was successfully inserted into the vector. The sequencing results are shown in Figures 4B, 5B. The sequencing results indicated that the *PKHD1*-wt and *PKHD1*-mut minigenes were successfully inserted into the pcMINI-C and pcDNA3.1 vectors, respectively. The sequence differences before and after the variant are shown in Figures 4D, 5D.

#### 3.4.2 *PKHD1* mRNA expression in the cells transfected with recombinant plasmids

To verify the effect of the c.2408-13C>G variant in *PKHD1* on pre-mRNA splicing, a minigene assay was performed. HeLa and 293T cells were transfected as previously described. Eight samples were collected 48 h after transfection. Schematic diagrams of the minigene construct are shown in Figures 4A, 5A.

The agarose gels showed no significant difference in the bands produced by the wt compared to the mut ones (Figures 4C, 5C), probably because the difference of 12 bp was too small. However, the difference could be seen in the sequencing results (Figures 4D, 5D). The wt band of the expected size was named band a, whereas the mut band was named band b (Figures 4C, 5C). The size of the pcMINI-C-*PKHD1*-wt band (a) was 601 bp, which was consistent with the expected size (Figure 4C). DNA sequencing results indicated that the wt minigene formed a normal mRNA comprising exon A (192 bp), exon 24 (185 bp), and exon 25 (123 bp) [Figure 4D (a)]. The intron c.2408-13C>G mutant minigene (pcMINI-C-*PKHD1*-mut) caused aberrant splicing, resulting in the retention of 12 bp on the right side of intron 23, and the splicing mode was exon A (192 bp), ▽intron 23 (12 bp), exon 24 (185 bp), and exon 25 (123 bp) [Figure 4D (b)]. The size of the pcDNA3.1*-PKHD1*-wt band (a) was 434 bp, which was consistent with the expected size (Figure 5C). Sequencing showed that the wt band a was spliced normally, and the splicing mode of the band was exon 23 (128 bp) to exon 24 (185 bp) [Figure 5D (a)]. The intron c.2408-13C>G mutant minigene (pcDNA3.1-*PKHD1*-mut) retained 12 bp to the right of intron 23. The splicing modes of the mut band b were exon 23 (128 bp), ▽intron 23 (12 bp), and exon 24 (185 bp) [Figure 5D (b)]. The sequencing results of the bands are shown in Figures 4D, 5D. These results showed that the *PKHD1-*mut minigenes produced alternative transcripts that were different from those of the *PKHD1*-wt minigenes in the two cell types. These results were consistent with those of the HSF, Varseak, and SpliceAI analyses.

## 4 Discussion

Ultrasonography has been routinely used for screening ARPKD (Sweeney and Avner, 1993-2019; Bergmann et al., 2018; Raina et al., 2021; Society for Maternal-Fetal Medicine and Swanson, 2021). In this study, prenatal ultrasonography demonstrated that the fetus had massively enlarged echogenic kidneys and oligohydramnios, but no liver lesions were found. Therefore, the fetus was highly suspected to be a patient with ARPKD. However, the prenatal diagnosis of ARPKD using fetal sonography can be unreliable, particularly during early pregnancy (Sweeney and Avner, 1993-2019; Zerres et al., 1998; Raina et al., 2021). Precise and unequivocal diagnosis of ARPKD requires genetic testing (Sweeney and Avner, 1993-2019; Bergmann et al., 2018; Groopman et al., 2018; Raina et al., 2021; Society for Maternal-Fetal Medicine and Swanson, 2021). Even so, considering the poor prognosis of pulmonary hypoplasia caused by severe oligohydramnios, the family herein decided to terminate the pregnancy without genetic result. After pregnancy termination, the parents chose genetic testing to seek the causes.

Trio-WES was used to explore the possible genetic causes of fetal abnormalities in the pedigree. Two variants of the *PKHD1* gene in the proband were detected in this study: (1) exon 58: c.9455del (p.N3152Tfs*10) in the father, and (2) intron 23: c.2408-13C>G in the mother. *PKHD1* is the most common pathogenic gene associated with ARPKD. A recent study indicated that the DAZ interacting zinc finger protein 1-like (*DZIP1L*) gene may be the second gene associated with ARPKD (Lu et al., 2017); however, more evidence is required for this to be definitively proven. ARPKD is caused by homozygous or compound heterozygous variants of the *PKHD1* gene (Onuchic et al., 2002; Ward et al., 2002). Variants of *PKHD1* are scattered throughout the gene, without evidence of clustering at specific sites (Bergmann et al., 2003; Bergmann et al., 2005a). There is no indication of a mutation hotspot (Bergmann et al., 2005a; Losekoot et al., 2005). Currently more than 500 pathogenic variants in *PKHD1* have been reported, and their variant types are diverse, including missense, nonsense, splicing, insertions, and deletions (Sweeney and Avner, 1993-2019; Adeva et al., 2006). At present, there have only been a few functional experiments on *PKHD1* gene mutation sites. Therefore, *in silico* predictions can provide an effective reference.

The exon variant (c.9455del) detected in this study has been previously reported (Cao et al., 2019). The c.9455del variant did not affect splicing, and the splicing pattern was consistent with that of the wt. However, this variant is a frameshift deletion that causes changes in the open reading frame and amino acid sequences. This variant has been evaluated as a disease-causing mutation in the Human Gene Mutation Database (HGMD) (Cao et al., 2019). The c.2408-13C>G variant was an intronic variant that has not been previously reported. It was not a classic splicing site (Cartegni et al., 2002); therefore, its clinical significance remained unknown. Thereafter, we used bioinformatic splice prediction tools to assess the possible effect of the c.2408-13C>G variant, which revealed that the variant might have a great impact on splice site function. Although intronic variants may have more harmful effects than exon variants, they have rarely been studied. To further clarify the pathogenicity of intronic variant c.2408-13C>G, we conducted functional studies.

It has been reported that a minigene experiment can produce splicing results reaching almost 100% similarity (van der Klift et al., 2015). Therefore, we performed RT-PCR splicing validation by constructing a minigene vector. Clinical interpretation of the splicing results of a genetic variant is complex. A variant is considered pathogenic when it causes frameshifts, major splicing aberrations, or in-frame insertions/deletions. The results of the minigene experiment *in vitro* showed that the intron c.2408-13C>G variant caused aberrant splicing, resulting in the retention of 12 bp in intron 23. And the results of the pcMINI-C and pcDNA3.1 vectors were the same. The cDNA after the variant was c.2407_2408insCTTCTCTCACAG. Moreover, the variant did not lead to a change in the subsequent reading frame. Therefore, our minigene splicing assay of the wt and mut constructs revealed that an aberrant splicing process was produced with the c.2408-13C>G variant at the mRNA level. According to this result, we hypothesized that the change in protein level was p. Ser802_Asp803insAlaSerLeuThr, which would lead to the insertion of four amino acid residues. The insertion of these four amino acid residues may change the structure and function of the protein. Functional validation demonstrated that the intron c.2408-13C>G variant caused aberrant splicing, which would subsequently lead to gene function impairment (strong pathogenic evidence, PS3), according to the ACMG criteria (Richards et al., 2015). Taken together, the evidence for the c.2408-13C>G variant was “PM2+PM3+PP3+PP4+PS3” and was updated to be a pathogenic variant. Changes in the protein levels were only hypothesized, and further studies are still required to confirm the role of the *PKHD1* gene variant (c.2408-13C>G) in ARPKD at the protein level.

Similar to this study, in suspected patients of other diseases, part of pathogenic gene variants are variants of unknown clinical significance (VUS) or likely pathogenic, which makes genetic counseling of patients and their families complicated. More importantly, in families who wish to have a healthy child, this causes difficulty in prenatal diagnosis. Therefore, functional studies for these variants are required. If the pathogenic variant occurs in an intron, RNA from the patient should be examined via RT-PCR analysis to establish whether the variant has any effect on splicing. However, it is usually difficult to timely keep samples from patients for RNA extraction. Alternatively, a variant in an intron can be examined by minigene splicing analysis (Bonnet et al., 2008; Thery et al., 2011). Splicing reporter minigenes have the following advantages: (1) no need to extract RNA from the patient; (2) analysis and quantification of the splicing outcome of a single mutant allele without the interference of the wt one; (3) high reproducibility of results. Herein, we successfully functionally characterized an intronic variant by minigene splicing. Two pathogenic variants were identified in this family member. At conception, each child of the parents will have a 25% chance of inheriting both pathogenic variants and being affected in the future. To have children unaffected by ARPKD, prenatal testing for future pregnancies or preimplantation genetic diagnosis (PGD) are possible options (Lau et al., 2010).

## 5 Conclusion

In this study, using minigene vector technology, we confirmed that the intron c.2408-13C>G variant can cause aberrant splicing, resulting in the retention of 12 bp in intron 23, which was a pathogenic site. This novel finding broadens the variant spectrum of the *PKHD1* gene and provides a basis for genetic counseling and diagnosis of ARPKD. Trio-WES is an effective high-throughput approach for the diagnosis of PKD.

## Data Availability

The datasets presented in this study can be found in the NCBI SRA database, accession number: PRJNA967496 (https://www.ncbi.nlm.nih.gov/sra/PRJNA967496).

## References

[B1] Abuín J. M. Pichel J. C. Pena T. F. Amigo J. (2015). BigBWA: Approaching the burrows-wheeler aligner to big data technologies. Bioinformatics 31 (24), 4003–4005. 10.1093/bioinformatics/btv506 26323715

[B2] Adeva M. El-Youssef M. Rossetti S. Kamath P. S. Kubly V. Consugar M. B. (2006). Clinical and molecular characterization defines a broadened spectrum of autosomal recessive polycystic kidney disease (ARPKD). Med. Baltim. 85 (1), 1–21. 10.1097/01.md.0000200165.90373.9a 16523049

[B3] Bergmann C. Senderek J. Sedlacek B. Pegiazoglou I. Puglia P. Eggermann T. (2003). Spectrum of mutations in the gene for autosomal recessive polycystic kidney disease (ARPKD/PKHD1). J. Am. Soc. Nephrol. 14 (1), 76–89. 10.1097/01.asn.0000039578.55705.6e 12506140

[B4] Bergmann C. Senderek J. Kupper F. Schneider F. Dornia C. Windelen E. (2004a). PKHD1 mutations in autosomal recessive polycystic kidney disease (ARPKD). Hum. Mutat. 23 (5), 453–463. 10.1002/humu.20029 15108277

[B5] Bergmann C. Senderek J. Schneider F. Dornia C. Kupper F. Eggermann T. (2004b). PKHD1 mutations in families requesting prenatal diagnosis for autosomal recessive polycystic kidney disease (ARPKD). Hum. Mutat. 23 (5), 487–495. 10.1002/humu.20019 15108281

[B6] Bergmann C. Kupper F. Dornia C. Schneider F. Senderek J. Zerres K. (2005a). Algorithm for efficient PKHD1 mutation screening in autosomal recessive polycystic kidney disease (ARPKD). Hum. Mutat. 25 (3), 225–231. 10.1002/humu.20145 15706593

[B7] Bergmann C. Senderek J. Windelen E. Kupper F. Middeldorf I. Schneider F. (2005b). Clinical consequences of PKHD1 mutations in 164 patients with autosomal-recessive polycystic kidney disease (ARPKD). Kidney Int. 67 (3), 829–848. 10.1111/j.1523-1755.2005.00148.x 15698423

[B8] Bergmann C. Guay-Woodford L. M. Harris P. C. Horie S. Peters D. J. M. Torres V. E. (2018). Polycystic kidney disease. Nat. Rev. Dis. Prim. 4 (1), 50. 10.1038/s41572-018-0047-y 30523303 PMC6592047

[B9] Bergmann C. (2017). Genetics of autosomal recessive polycystic kidney disease and its differential diagnoses. Front. Pediatr. 5, 221. 10.3389/fped.2017.00221 29479522 PMC5811498

[B10] Bonnet C. Krieger S. Vezain M. Rousselin A. Tournier I. Martins A. (2008). Screening BRCA1 and BRCA2 unclassified variants for splicing mutations using reverse transcription PCR on patient RNA and an *ex vivo* assay based on a splicing reporter minigene. J. Med. Genet. 45 (7), 438–446. 10.1136/jmg.2007.056895 18424508

[B11] Cao Q. Zhang W. Ge J. Sun D. Feng Q. Li C. (2019). Prenatal diagnosis and genetic counseling in two pedigrees affected with infantile polycystic kidney disease due to PKHD1 gene mutations. Zhonghua Yi Xue Yi Chuan Xue Za Zhi 36 (8), 765–768. 10.3760/cma.j.issn.1003-9406.2019.08.003 31400123

[B12] Cartegni L. Chew S. L. Krainer A. R. (2002). Listening to silence and understanding nonsense: Exonic mutations that affect splicing. Nat. Rev. Genet. 3 (4), 285–298. 10.1038/nrg775 11967553

[B13] Dong C. Yu B. (2011). Mutation surveyor: An *in silico* tool for sequencing analysis. Methods Mol. Biol. 760, 223–237. 10.1007/978-1-61779-176-5_14 21780000

[B14] Groopman E. E. Rasouly H. M. Gharavi A. G. (2018). Genomic medicine for kidney disease. Nat. Rev. Nephrol. 14 (2), 83–104. 10.1038/nrneph.2017.167 29307893 PMC5997488

[B15] Gross M. B. (2016). Personal communication. Baltimore, Md.

[B16] Guay-Woodford L. M. Desmond R. A. (2003). Autosomal recessive polycystic kidney disease: The clinical experience in north America. Pediatrics 111 (5), 1072–1080. 10.1542/peds.111.5.1072 12728091

[B17] Guay-Woodford L. M. Muecher G. Hopkins S. D. Avner E. D. Germino G. G. Guillot A. P. (1995). The severe perinatal form of autosomal recessive polycystic kidney disease maps to chromosome 6p21.1-p12: Implications for genetic counseling. Am. J. Hum. Genet. 56 (5), 1101–1107. 7726165 PMC1801440

[B18] Guay-Woodford L. M. Bissler J. J. Braun M. C. Bockenhauer D. Cadnapaphornchai M. A. Dell K. M. (2014). Consensus expert recommendations for the diagnosis and management of autosomal recessive polycystic kidney disease: Report of an international conference. J. Pediatr. 165 (3), 611–617. 10.1016/j.jpeds.2014.06.015 25015577 PMC4723266

[B19] Gunay-Aygun M. Avner E. D. Bacallao R. L. Choyke P. L. Flynn J. T. Germino G. G. (2006). Autosomal recessive polycystic kidney disease and congenital hepatic fibrosis: Summary statement of a first national institutes of health/office of rare diseases conference. J. Pediatr. 149 (2), 159–164. 10.1016/j.jpeds.2006.03.014 16887426 PMC2918414

[B20] Hartung E. A. Guay-Woodford L. M. (2014). Autosomal recessive polycystic kidney disease: A hepatorenal fibrocystic disorder with pleiotropic effects. Pediatrics 134 (3), e833–e845. 10.1542/peds.2013-3646 25113295 PMC4143997

[B21] Horie S. Mochizuki T. Muto S. Hanaoka K. Fukushima Y. Narita I. (2016). Evidence-based clinical practice guidelines for polycystic kidney disease 2014. Clin. Exp. Nephrol. 20 (4), 493–509. 10.1007/s10157-015-1219-7 27095364 PMC4956721

[B22] Lau E. C. Janson M. M. Roesler M. R. Avner E. D. Strawn E. Y. Bick D. P. (2010). Birth of a healthy infant following preimplantation PKHD1 haplotyping for autosomal recessive polycystic kidney disease using multiple displacement amplification. J. Assist. Reprod. Genet. 27 (7), 397–407. 10.1007/s10815-010-9432-5 20490649 PMC2922704

[B23] Losekoot M. Haarloo C. Ruivenkamp C. White S. J. Breuning M. H. Peters D. J. (2005). Analysis of missense variants in the PKHD1-gene in patients with autosomal recessive polycystic kidney disease (ARPKD). Hum. Genet. 118 (2), 185–206. 10.1007/s00439-005-0027-7 16133180

[B24] Lu H. Galeano M. C. R. Ott E. Kaeslin G. Kausalya P. J. Kramer C. (2017). Mutations in DZIP1L, which encodes a ciliary-transition-zone protein, cause autosomal recessive polycystic kidney disease. Nat. Genet. 49 (7), 1025–1034. 10.1038/ng.3871 28530676 PMC5687889

[B25] Minton J. A. Flanagan S. E. Ellard S. (2011). Mutation surveyor: Software for DNA sequence analysis. Methods Mol. Biol. 688, 143–153. 10.1007/978-1-60761-947-5_10 20938837

[B26] Onuchic L. F. Furu L. Nagasawa Y. Hou X. Eggermann T. Ren Z. (2002). PKHD1, the polycystic kidney and hepatic disease 1 gene, encodes a novel large protein containing multiple immunoglobulin-like plexin-transcription-factor domains and parallel beta-helix 1 repeats. Am. J. Hum. Genet. 70 (5), 1305–1317. 10.1086/340448 11898128 PMC447605

[B27] Raina R. Chakraborty R. Sethi S. K. Kumar D. Gibson K. Bergmann C. (2021). Diagnosis and management of renal cystic disease of the newborn: Core curriculum 2021. Am. J. Kidney Dis. 78 (1), 125–141. 10.1053/j.ajkd.2020.10.021 33418012

[B28] Richards S. Aziz N. Bale S. Bick D. Das S. Gastier-Foster J. (2015). Standards and guidelines for the interpretation of sequence variants: A joint consensus recommendation of the American College of medical genetics and genomics and the association for molecular Pathology. Genet. Med. 17 (5), 405–424. 10.1038/gim.2015.30 25741868 PMC4544753

[B29] Robinson J. T. Thorvaldsdóttir H. Winckler W. Guttman M. Lander E. S. Getz G. (2011). Integrative genomics viewer. Nat. Biotechnol. 29 (1), 24–26. 10.1038/nbt.1754 21221095 PMC3346182

[B30] Society for Maternal-Fetal Medicine (Smfm) Swanson K. (2021). Autosomal recessive polycystic kidney disease. Am. J. Obstet. Gynecol. 225 (5), B7–B8. 10.1016/j.ajog.2021.06.038 34507795

[B31] Sweeney W. E. Avner E. D. (1993-2019). "Polycystic kidney disease, autosomal recessive. 2001 jul 19 [Updated 2019 Feb 14]," in GeneReviews^R^ , eds. Adam M. P. Everman D. B. Mirzaa G. M. Pagon R. A. Wallace S. E. Bean L. J. H. Seattle (WA): University of Washington. 20301501

[B32] Thery J. C. Krieger S. Gaildrat P. Revillion F. Buisine M. P. Killian A. (2011). Contribution of bioinformatics predictions and functional splicing assays to the interpretation of unclassified variants of the BRCA genes. Eur. J. Hum. Genet. 19 (10), 1052–1058. 10.1038/ejhg.2011.100 21673748 PMC3190263

[B33] Thorvaldsdóttir H. Robinson J. T. Mesirov J. P. (2013). Integrative genomics viewer (IGV): High-performance genomics data visualization and exploration. Brief. Bioinform 14 (2), 178–192. 10.1093/bib/bbs017 22517427 PMC3603213

[B34] van der Klift H. M. Jansen A. M. van der Steenstraten N. Bik E. C. Tops C. M. Devilee P. (2015). Splicing analysis for exonic and intronic mismatch repair gene variants associated with Lynch syndrome confirms high concordance between minigene assays and patient RNA analyses. Mol. Genet. Genomic Med. 3 (4), 327–345. 10.1002/mgg3.145 26247049 PMC4521968

[B35] Wang K. Li M. Hakonarson H. (2010). Annovar: Functional annotation of genetic variants from high-throughput sequencing data. Nucleic Acids Res. 38 (16), e164. 10.1093/nar/gkq603 20601685 PMC2938201

[B36] Ward C. J. Hogan M. C. Rossetti S. Walker D. Sneddon T. Wang X. (2002). The gene mutated in autosomal recessive polycystic kidney disease encodes a large, receptor-like protein. Nat. Genet. 30 (3), 259–269. 10.1038/ng833 11919560

[B37] Writing Group For Practice Guidelines For Diagnosis And Treatment Of Genetic Diseases Medical Genetics Branch Of Chinese Medical Association Xu D. Mei C. (2020). Clinical practice guidelines for polycystic kidney diseases. Zhonghua Yi Xue Yi Chuan Xue Za Zhi 37 (3), 277–283. 10.3760/cma.j.issn.1003-9406.2020.03.009 32128744

[B38] Zerres K. Mucher G. Becker J. Steinkamm C. Rudnik-Schoneborn S. Heikkila P. (1998). Prenatal diagnosis of autosomal recessive polycystic kidney disease (ARPKD): Molecular genetics, clinical experience, and fetal morphology. Am. J. Med. Genet. 76 (2), 137–144. 10.1002/(SICI)1096-8628(19980305)76:2<137::AID-AJMG6>3.3.CO;2-O 9511976

